# The Role of the Embodiment Disturbance in the Anorexia Nervosa Psychopathology: A Network Analysis Study

**DOI:** 10.3390/brainsci9100276

**Published:** 2019-10-15

**Authors:** Giammarco Cascino, Giovanni Castellini, Giovanni Stanghellini, Valdo Ricca, Emanuele Cassioli, Valeria Ruzzi, Palmiero Monteleone, Alessio Maria Monteleone

**Affiliations:** 1 Department of Medicine, Surgery and Dentistry ‘Scuola Medica Salernitana’, Section of Neurosciences, University of Salerno, 84081 Salerno, Italy; 2 Psychiatry Unit, Department of Health Sciences, University of Florence, 50139 Florence, Italy; 3 Department of Psychological, Humanistic and Territorial Sciences, ‘G. d’Annunzio’ University, 66100 Chieti, Italy; 4 ‘D. Portales’ University, 8320000 Santiago, Chile; 5 Department of Psychiatry, University of Campania “Luigi Vanvitelli”, 80138 Naples, Italy

**Keywords:** anorexia nervosa, embodiment, network analysis, body image, body awareness

## Abstract

Anorexia Nervosa (AN) is characterized by body image distortion. From a phenomenological perspective, body image disturbance has been associated with a more profound disturbance encompassing disorders of the way persons experience their own body. The aim of this study was to disentangle the complex dynamics that connect the experience of one’s own body and self-identity to the psychopathological features of AN by applying a network analysis. Fifty-seven patients with AN restrictive subtype and 27 with AN binge–purging subtype participated in the study. Eating Disorders Inventory-2 and Identity and Eating Disorders subscores, measuring the embodiment dimensions, were included in the network. Two of the main dimensions of embodiment—feeling extraneous from one’s own body and feeling oneself through objective measures—were the nodes with the highest strength together with interoceptive awareness (IA). IA was a node included in several pathways connecting embodiment dimensions with most of the AN psychopathological dimensions. The centrality of the embodiment disorder suggests the importance of considering the body image disturbance in people with AN as resulting from their difficulty in experiencing inner states and as a tool to build its own self. This assumption may orient therapeutic interventions.

## 1. Introduction

Anorexia nervosa (AN) is a psychiatric syndrome that occurs mostly in adolescence and is characterized by severe disturbances in eating behaviour, as dietary restriction or starvation, leading to underweight. The core symptoms of AN also include body image distortion, that is feeling fat while underweight, and disturbances in interoceptive awareness [1,2]. According to the Fairburn and Harrison’s model [3], it has been suggested that overvaluation of body shape and weight or the ability to control them play a significant role in the onset and maintenance of AN psychopathology [4,5]. From a phenomenological perspective, the body image disturbance has been associated with a more profound disturbance encompassing disorders of the way persons experience their own body (i.e., embodiment) and shape their personal identity. To date, although weight and shape concern represents a core aspect of AN, few studies have adopted this particular way of conceptualizing body image disturbance in eating disorder (ED) psychopathology.

On the basis of this theoretical background, Stanghellini et al. [6] developed and validated a self-reported questionnaire, the IDentity and EAting disorders (IDEA), applying the Sartrean concept of the experience of one’s own body as an object being looked at by another, which overcomes the traditional phenomenological distinction between lived body (Leib) and physical body (Koerper) [6]. According to this position, the reason why persons with AN overvalue their body shape and weight can be better understood as a specific disorder of lived corporeality, and more specifically as the predominance of one dimension of embodiment, namely the ‘lived-body-for-others’. Moreover, feeling extraneous from one’s own body has been demonstrated to be the experience that discriminates most between people with EDs and nonclinical subjects, and it has been proposed that the increased trend to perceive themselves from an external perspective is the way to cope with identity problems [7]. Thus, abnormal eating behaviours in people with AN may be conceived as strategies to feel themselves for people who are unable to feel themselves cenesthetically [7]. Recently, it has been also proved that the disorder of embodiment is associated with insecure attachment experiences and mediates the relationships between avoidant attachment and ED psychopathology [8]. In line with these findings, Eshkevari et al. [9] demonstrated through an experimental task that somatosensory information processing of the body may be altered in people with AN and that these alterations partially persist after recovery [10]. Furthermore, it has been shown that interoceptive sensitivity predicts the ability to represent body image [11], and body awareness has been considered the basis for the embodiment disturbances in EDs [12]. However, even though the centrality of interoceptive deficits in AN has been highlighted [13,14,15], body image disturbance in AN was mostly investigated through its cognitive-affective component [16]. As a consequence, the interplay between embodiment disturbances and the core symptoms of AN needs to be investigated.

In order to investigate the relationships between embodiment dimensions and AN core psychopathology, we applied the network analysis (NA) approach. Network theory defines a disorder as a constellation of symptoms, which activate one another promoting a state of prolonged and self-sustaining symptom activation [17,18]. This approach allows the disentanglement of the complex interaction between symptoms. Moreover, the NA identifies the strength through which a variable included in the network (a node) is connected to the other ones, that is named network centrality [19]. It has been shown that the most central nodes of the networks are able to predict the outcome in people with EDs, thus representing possible therapeutic targets [20,21,22].

To the best of our knowledge, no study has used this methodological approach to investigate the relationships between variables of embodiment dimension and AN psychopathology. Therefore, the first aim of this study was to gain a deeper insight into the complex dynamics that connect the experience of one’s own body and self-identity to the psychopathological features of AN by applying a NA. Our first hypothesis was that dimensions of the embodiment should present the highest centrality in the network. This hypothesis is grounded on the suggested theory that dysfunctional eating behaviours should be considered as a kind of epiphenomenon of a more profound psychopathological core associated with disorders of embodiment, thus representing coping strategies to face the unstable sense of one’s self deriving from the reduced ability in perceiving emotional and bodily experiences [23]. Then, we also hypothesized that the interoceptive awareness would play a role in connecting embodiment deficits and AN symptoms, given the proved association of interoceptive awareness with both the embodiment disturbances [12] and the ED psychopathology [2,14].

## 2. Materials and Methods

### 2.1. Procedure

Consecutive patients attending the Eating Disorder Centre of the Department of Psychiatry at the University of Campania “Luigi Vanvitelli” were included in the study if they met the following criteria: (a) female gender; (b) age ≥18; (c) current diagnosis of AN or atypical AN according to the fifth edition of Diagnostic and Statistical Manual of Mental Disorders (DSM-5) criteria; (d) absence of current Axis I comorbid psychiatric disorders; and (e) willingness to cooperate in the experimental procedures and to sign a written informed consent. Trained psychiatrists using the Structured Clinical Interview for DSM-5 Disorders—Research Version [24] made diagnostic assessment and collected sociodemographic and clinical data. The self-report questionnaires were completed before starting specific treatment programs. The study was approved by the Institutional Board of the “Department of Mental Health, Physical Health, and Preventive Medicine” of the University of Campania “Luigi Vanvitelli”, Naples, Italy.

### 2.2. Clinical Assessment

The Eating Disorders Inventory-2 (EDI-2) [25] is a self-report questionnaire, which evaluates ED symptomatology and psychopathology. The questionnaire includes 11 subscales: ineffectiveness (IN), which assesses feelings of inadequacy, worthlessness, and having no control over one’s own life; social insecurity (SI), which explores the fear of social situations; drive to thinness (DT), which investigates the excessive concerns with weight, dieting, and fear of weight gain; interoceptive awareness (IA), which evaluates the ability to discriminate feelings and the sensations of hunger and satiety; maturity fear (MF), which assesses the fear to address the demands of adult life; body dissatisfaction (BD), which measures the dissatisfaction with one’s physical appearance; perfectionism (P), which evaluates the dissatisfaction with anything less than perfect; interpersonal distrust (ID), which explores the reluctance to form close relationships; impulsivity (I), which investigates the ability to regulate impulsive behaviour; bulimia (BU), which assesses the presence and frequency of binge–purging episodes; and asceticism (ASC), which evaluates the pursuit of spiritual ideals, such as self-discipline, self-denial, and the control of the needs of one’s body.

The IDEA is a self-report scale that assesses abnormalities in lived corporeality and personal identity [6] and consists of 23 items divided into four subscales: Feeling oneself through the gaze of the other and defining oneself through the evaluation of the other (GEO; e.g., ‘I cannot stand not to know what the others think of me’), feeling oneself through objective measures (OM; e.g., ‘Having control over my weight means having control over the possible changes that happen in my body’), feeling extraneous from one’s own body (EB; e.g., ‘I see myself out of focus, I do not feel myself’), feeling oneself through starvation (S; e.g., ‘Eating according to my own rules is the only way to feel myself’).

### 2.3. Network Analysis and Statistical Procedures

NA was performed through R, version 3.4.4 (R core Team, Vienna, Austria), using qgraph package according to the methodology described by Epskamp et al. [26]. A partial correlation network was estimated. A network is composed by nodes representing measured variables and by edges representing that two variables are not independent after conditioning on all variables in the dataset. These edges have a thickness, or edge weight, which are the partial correlation coefficients. Whenever there is no edge between two nodes, the partial correlation coefficient is zero and it means that two variables are independent after controlling for all other variables in the network [27]. In order to limit the number of spurious connections, we applied “least absolute shrinkage and selection operator” (LASSO) regularization [28] that shrinks small partial correlations, setting them to zero. The Extended Bayesian Information Criterion (EBIC) [29], a parameter that sets the degree of regularization/penalty applied to sparse correlations, was set to 0.5 in this analysis, as suggested by Foygel and Drton [30] to avoid most spurious edges. 

Nodes are characterized by centrality measures that are a way of assessing the importance of nodes in the network, with higher values indicating that nodes are more important [31]. There are three centrality indices: strength, betweenness, and closeness. In particular, the node strength refers to the sum of the absolute edge weights between a focal node and all other nodes to which it is connected in the network [32].

In order to assess the accuracy of the obtained psychological network, following the Epskamp’s recommendations [32], first we estimated confidence intervals (CIs) on the edge-weights calculated by means of “nonparametric” bootstrapping (nboots = 2500). We assessed the stability of centrality indices through the re-estimating of the network and re-calculation of centrality indices after dropping growing percentages of cases, followed by the calculation of the correlation stability (CS) coefficient. The CS is the maximum proportion of population that can be dropped with re-calculated indices correlating at least 0.7 with indices of the original sample. It is recommended that the CS should not be below 0.25 [32].

A two-tailed t-test was performed to test possible significant differences in demographic and clinical variables between diagnostic groups. Resulting p-values were corrected using the Bonferroni’s criterion for multiple comparisons.

## 3. Results

### 3.1. Sample Characteristics

Eighty-four patients participated in the study. Fifty-seven received diagnoses of AN restrictive subtype and 27 of AN binge–purging subtype. Demographic and clinical characteristics of the sample are reported in Table 1. After applying correction for multiple comparisons, no significant differences were detected between groups in demographic and clinical variables, except for EDI-2 BU subscores.

### 3.2. Network Analysis

**Figure 1:**
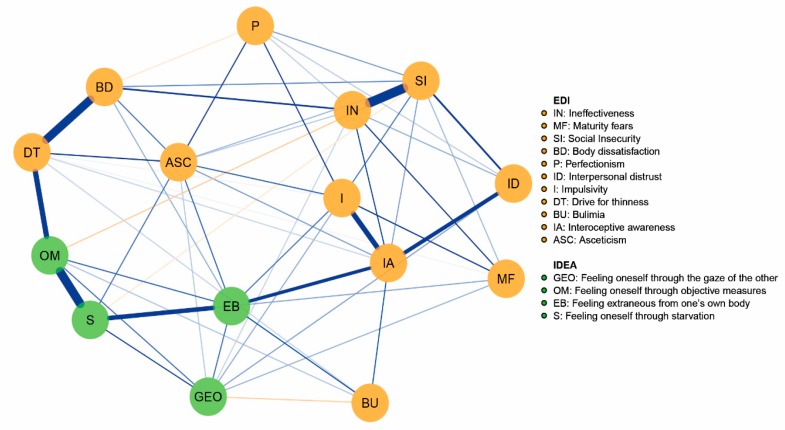
Estimated network of the sample population.

**Figure 2:**
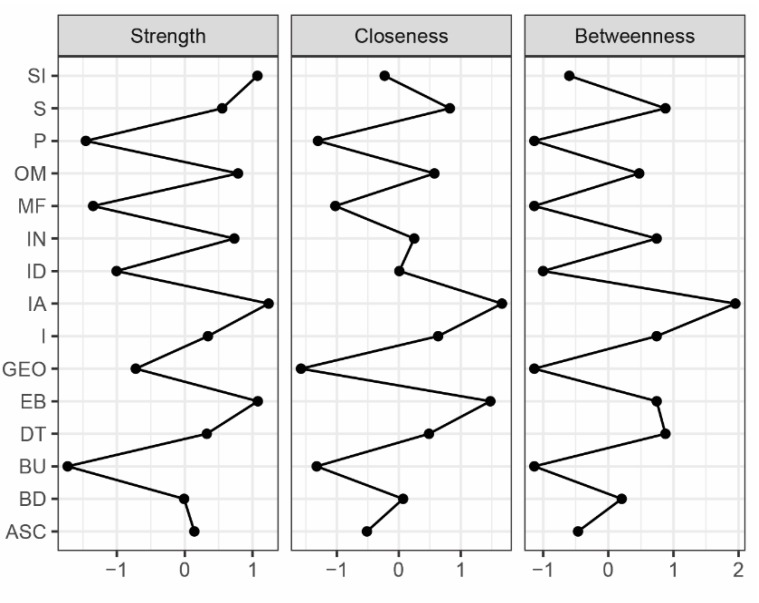
Plot of centrality indices of the network depicting the strength, closeness, and betweenness of each node. GEO, feeling oneself through the gaze of the other; OM, feeling oneself through objective measures; EB, feeling extraneous from one’s own body; S, feeling oneself through starvation; EDI, Eating Disorders Inventory; IN, ineffectiveness; MF, maturity fear; SI, social insecurity; BD, body dissatisfaction; P, perfectionism; ID, interpersonal distrust; I, impulsivity; DT, drive for thinness; BU, bulimia; IA, interoceptive awareness; ASC, asceticism.

The network is reported in Figure 1. The blue edges indicate positive partial correlation, while the orange lines show the negative ones. The centrality indices of the variables included in the network are plotted in Figure 2. The correlation stability coefficients are 0.285 for strength and closeness that are above the recommended cutoff value of 0.25 and below 0.25 for betweenness. The accuracy of centrality indices is shown in Supplementary Figure S1.

The nodes with the highest strength centrality are EDI-2 IA (M = 1.28), IDEA EB (M = 1.23), EDI-2 SI (M = 1.23), and IDEA OM (M = 1.14). The strongest connections of EDI-2 IA are with I (0.26) and ID (0.24) among EDI-2 variables and EB (0.24) among embodiment measures. The strongest connections of IDEA EB are with IDEA S (0.29), EDI-2 IA, and EDI-2 I (0.13). IDEA OM is strongly connected with IDEA S (0.42) and EDI-2 DT (0.31), while EDI-2 SI is associated with EDI-2 IN (0.44).

The bootstrapped confidence intervals of estimated edge-weights are reported in Supplementary Figure S2.

## 4. Discussion

This is the first study that investigated the relationships between embodiment dimensions and core psychopathology in people with AN through the NA approach. The hypothesized centrality of embodiment for AN psychopathology was only partially confirmed, since, two of the main dimensions of embodiment—namely feeling extraneous from one’s own body and feeling oneself through objective measures—were the nodes with the highest strength after interoceptive awareness and before social insecurity. Our second study hypothesis was confirmed, since interoceptive awareness was a node included in a number of pathways connecting embodiment dimensions with most of the AN psychopathological variables. Finally, an unexpected direct strong connection between feeling oneself through objective measures and drive to thinness was also revealed.

Our partial correlations network allows the assessment of the connections between each pair of variables taking into account the effect of all the other variables included in the network. This analysis showed that feeling extraneous from one’s own body and feeling oneself through objective measures were the nodes with the highest centrality strength and, thus, the nodes most directly connected to the other nodes in the network [27]. The activation of a node may cause the development of the connected symptoms; consequently, the most central nodes have been conceptualized as risk factors for developing further symptoms [33], in our case, AN symptoms. Although the high centrality of a node has been argued to be a possible effect of connections with other symptoms [34] and the cross-sectional nature of our data does not allow definitive conclusions regarding causality to be drawn, embodiment dimensions may represent targets for psychotherapeutic intervention. In accordance with the network theory [17], previous studies highlighted the prognostic value of the central nodes in EDs. Olatunji et al. [20] highlighted ineffectiveness and interoceptive awareness as central nodes and demonstrated that ineffectiveness predicted body mass index (BMI) and depressive symptoms at discharge in an inpatient sample with EDs. Elliott et al. [21] highlighted the prognostic value of the central nodes (feeling fat, uncomfortable with one’s own body, the desire to lose weight) with respect to the outcomes of the treatment, defined as recover and clinical impairment at 12 months, in people with AN. Furthermore, avoidance of emotional feelings has been recently identified as an important therapeutic predictive factor in people with AN [35].

Among the most central nodes, the feeling extraneous from one’s own body node represents a measure of an individual’s alienation from his own body and emotions [6]. This peculiar disturbance in bodily feelings (as e.g., seeing oneself out of focus, fuzzy or not being able to feel oneself) may represent a core phenomenon in patients with AN. In line with this assumption, feeling extraneous from one’s own body was identified as an intermediate phenotype able to identify people who are more prone to develop full-blown EDs [7]. Furthermore, a NA study conducted on adolescents with short duration of AN and including several general and specific psychopathology measures, showed that personal alienation was one of the nodes with the highest centrality [36].

The OM node represents a measure of feeling oneself through objective measures. The centrality of this variable corroborates the psychosocial construct “public self-consciousness” [37], which highlights the importance of all qualities of the self that are perceived from an external perspective as central to construct the person’s identity in people with EDs. An unexpected finding of our NA was the presence of a strong connection of the OM node with drive to thinness. This is in line with previous studies describing the abnormal eating behaviour as a tool for achieving a new identity [38,39]. Indeed, even though the cross-sectional design of the study does not allow the ascertainment of a cause–consequence relationship between variables, the present results seem to confirm the proposed model for development of specific psychopathology in AN. Symptoms such as drive to thinness should be considered as a kind of dysfunctional coping strategy in patients with AN to cope with the sense of extraneousness toward body cenesthesis and emotions. In line with this hypothesis, the pattern of the present NA seems to confirm that the embodiment disturbance may be associated with pathological behaviours with the mediation of a desired lifestyle characterized by abstinence from sensual pleasures and severe discipline (the asceticism), which represents a possible strategy to compensate the need for self-definition.

Our secondary hypothesis was that interoceptive awareness played a role in connecting embodiment dimensions and the core psychopathology of AN. Interoceptive awareness was the node with the highest strength among the AN psychopathology nodes and showed strong partial correlations with feeling extraneous from one’s own body, interpersonal distrust, and impulsivity. A decreased interoceptive awareness is largely acknowledged in ED patients [40] and it has been related to body image distortion [41,42], decision-making abilities, and embodiment disturbances [12]. Interoceptive awareness was the node with the highest score in the closeness centrality index: This confirms its potential role in mediating the connections between the embodiment dimensions and the AN psychopathology. In accordance with this finding, interoceptive awareness was found to be a bridge symptom between self-esteem problems and the ED core symptoms in adolescents with AN [35]. Moreover, the network graph (Figure 1) indicates that interoceptive awareness mediates strongly the connections between feeling extraneous from one’s own body and impulsivity or interpersonal distrust. Previous studies found significant associations between all embodiment dimensions and ED core psychopathology but not with general psychiatric symptoms in people with EDs [6]. These pathways may represent potential targets for therapeutic interventions, as suggested by the network theory [17]. Moreover, our findings confirm the association between ED core symptoms and embodiment dimensions but also suggest, for the first time, that the most central embodiment constructs, i.e., feeling oneself through objective measures and feeling extraneous from one’s own body, are specifically associated with drive to thinness and interoceptive awareness while controlling for the effect of all ED core symptoms. 

The main strength of this study is the employment of the NA approach which, in spite of the relatively small size of our sample, showed acceptable values of reproducibility. The NA makes it possible to assess connections between each pair of nodes taking into account all other variables included in the network; therefore, our study provides, for the first time, a novel interpretation of the complex dynamics between embodiment dimensions and AN psychopathology. 

There are also some limitations that need to be taken into account. First, the sample size is relatively small and does not permit the analysis to be performed in each AN subtype. Indeed, the differences in the clinical presentation of people with different AN subtypes may suggest possible discrepancies between the network structure of each subtype of AN, thus affecting the generalization of present findings. On the other hand, no significant differences in the psychopathological variables were detected between the diagnostic subtypes in our sample, except for bulimic behaviours. Second, the cross-sectional design does not allow us to completely clarify causality of associations between embodiment dimensions and specific psychopathology. Indeed, it has been suggested that the high centrality of a node may be the effect of relationships with other nodes [34]. Thus, studies with longitudinal or experimental design are needed to better understand the causal dynamics between embodiment and AN psychopathology and to observe whether the maintenance of an embodiment disorder can impair clinical recovery or promote phenomena such as the diagnostic crossover. Finally, there is a lack of specific measures regarding social cognition, decision making abilities, and emotion regulation difficulties that may be worth including in the evaluation of connections between embodiment disorders and AN psychopathology.

### Clinical Implications

Overall, our findings support the idea that embodiment dimensions, especially the alienation from own body and emotions and the definition through objective measures, may be core features of AN psychopathology. More than half a century ago, Hilde Bruch [13] was the first to define AN as a “self”-disorder in which developmental problems of the organization of the self are manifested in aberrant body perception and inefficient interpretation of bodily sensations and inner feelings. She described the “interoceptive confusion” as part of a more complex identity disorder and conceived the drive to a thin body as a “camouflage” for other underlying problems (i.e., confusion of emotional states, control of bodily sensations, fear, or social disapproval). Our findings are in line with these observations. Thus, providing a new conceptualization of the body image disturbance may represent a novel opportunity for the psychotherapeutic treatments. This is worth taking into account on the light of either the treatment predictive value of body image distortion [43,44] or the lack of successful interventions in the treatment of this psychopathological feature [16]. In accordance with the network theory, our findings suggest that a correction of body misperception may be an essential precondition for recovery from AN.

Besides identity definition, the embodiment disorder plays a role in the interactions with others. Indeed, the reduced trend to perceive the body from the inner perspective leads to the perception of the others as the mirror in which one can see oneself and feel oneself. This may promote social cognition problems, ranging from mentalizing and empathy problems to heightened perception of potential social threats, which are so common in AN and should represent important treatment targets [45,46,47]. Future studies are needed to confirm this hypothesis, although evidence of a relationship between insecure intimate interactions and embodiment problems have been provided in people with EDs [8].

However, present findings suggest psychotherapeutic interventions to focus on the self-meaning of the eating symptoms. This refers to the possibility of assessing the interpersonal context which precedes abnormal eating behaviours in order to identify emotional problems and bodily sensations and of developing different coping strategies. Although specific treatment protocols are lacking, the use of interventions aiming to improve inner state recognition, such as the mindfulness-based stress reduction therapy introduced for people with depression [48], may be suggested.

## 5. Conclusions

In conclusion, the centrality of the embodiment disorder suggests to clinicians the importance of considering the body image disturbance as a tool to build its own self for people with AN. This assumption may orient therapeutic efforts.

## Figures and Tables

**Table 1:** Demographic and clinical characteristics of the whole sample and diagnostic subtype groups (means ± standard deviations).

	Whole Sample (*n* = 84)	Restricting Subtype (*n* = 57)	Binge-Purging Subtype (*n* = 27)
Age (years)	27.04 ± 8.80	26.33 ± 8.19	28.76 ± 10.09
BMI (kg/m^2^)	16.96 ± 1.87	16.77 ± 1.87	17.24 ± 1.78
Age at Onset	17.58 ± 4.93	17.48 ± 4.92	17.64 ± 4.95
Illness Duration	8.94 ± 8.85	8.22 ± 7.94	8.73 ± 8.75
IDEA Total	2.08 ± 1.07	1.98 ± 1.10	2.34 ± 0.97
IDEA GEO	1.83 ± 1.04	1.84 ± 1.07	1.85 ± 0.99
IDEA OM	2.38 ± 1.28	2.22 ± 1.36	2.73 ± 1.05
IDEA EB	1.91 ± 1.27	1.73 ± 1.29	2.38 ± 1.07
IDEA S	2.36 ± 1.25	2.23 ± 1.33	2.66 ± 1.03
EDI IN	12.40 ± 8.61	11.35 ± 8.65	15.00 ± 8.18
EDI MF	10.19 ± 6.67	10.11 ± 6.37	10.54 ± 7.47
EDI SI	8.74 ± 4.49	8.60 ± 4.52	9.38 ± 4.21
EDI BD	13.36 ± 7.93	12.61 ± 8.1	15.27 ± 7.39
EDI P	7.13 ± 4.82	6.93 ± 5.04	7.65 ± 4.45
EDI ID	7.94 ± 4.95	8.00 ± 5.11	8.04 ± 4.62
EDI IR	8.05 ± 7.36	7.25 ± 6.83	9.96 ± 8.35
EDI DT	12.51 ± 7.69	11.61 ± 8.08	14.81 ± 6.30
EDI BU	3.15 ± 4.96	1.30 ± 2.38	7.35 ± 6.51 *
EDI IA	12.60 ± 8.37	11.25 ± 8.09	16.00 ± 8.01
EDI ASC	7.54 ± 5.67	6.75 ± 4.92	9.38 ± 6.86

BMI, body mass index; IDEA, IDentity and EAting disorders; GEO, feeling oneself through the gaze of the other; OM, feeling oneself through objective measures; EB, feeling extraneous from one’s own body; S, feeling oneself through starvation; EDI, Eating Disorders Inventory; IN, ineffectiveness; MF, maturity fear; SI, social insecurity; BD, body dissatisfaction; P, perfectionism; ID, interpersonal distrust; I, impulsivity; DT, drive for thinness; BU, bulimia; IA, interoceptive awareness; ASC, asceticism.* vs. ANR *p* < 0.001.

## Supplementary Materials

The following are available at https://www.mdpi.com/2076-3425/9/10/276/s1, **Figure S1**. Average correlation between centrality indices of the networks sampled with persons dropped and the original sample. **Figure S2**. Bootstrapped confidence intervals of edge-weights in the estimated network.

## References

[1] Garner D.M., Olmstead M.P., Polivy J. (1983). Development and validation of a multidimensional eating disorder inventory for anorexia nervosa and bulimia. Int. J. Eat. Disord..

[2] Gaudio S., Brooks S.J., Riva G. (2014). Nonvisual Multisensory Impairment of Body Perception in Anorexia Nervosa: A Systematic Review of Neuropsychological Studies. PLoS ONE.

[3] Fairburn C.G., Cooper Z., Shafran R. (2003). Cognitive behaviour therapy for eating disorders: A “transdiagnostic” theory and treatment. Behav. Res. Ther..

[4] Dalle Grave R., Calugi S., Marchesini G. (2008). Compulsive exercise to control shape or weight in eating disorders: Prevalence, associated features, and treatment outcome. Compr. Psychiatry.

[5] Ricca V., Castellini G., Lo Sauro C., Mannucci E., Ravaldi C., Rotella F., Faravelli C. (2010). Cognitive-Behavioral Therapy for Threshold and Subthreshold Anorexia Nervosa: A Three-Year Follow-Up Study. Psychother. Psychosom..

[6] Stanghellini G., Castellini G., Brogna P., Faravelli C., Ricca V. (2012). Identity and eating disorders (IDEA): A questionnaire evaluating identity and embodiment in eating disorder patients. Psychopathology.

[7] Stanghellini G., Trisolini F., Castellini G., Ambrosini A., Faravelli C., Ricca V. (2015). Is Feeling Extraneous from One’s Own Body a Core Vulnerability Feature in Eating Disorders?. Psychopathology.

[8] Monteleone A.M., Castellini G., Ricca V., Volpe U., De Riso F., Nigro M., Zamponi F., Mancini M., Stanghellini G., Monteleone P. (2017). Embodiment Mediates the Relationship between Avoidant Attachment and Eating Disorder Psychopathology. Eur. Eat. Disord. Rev..

[9] Eshkevari E., Rieger E., Longo M.R., Haggard P., Treasure J. (2012). Increased plasticity of the bodily self in eating disorders. Psychol. Med..

[10] Eshkevari E., Rieger E., Longo M.R., Haggard P., Treasure J. (2014). Persistent body image disturbance following recovery from eating disorders. Int. J. Eat. Disord..

[11] Tsakiris M., Jimenez A.T., Costantini M. (2011). Just a heartbeat away from one’s body: Interoceptive sensitivity predicts malleability of body-representations. Proc. R. Soc. B Biol. Sci..

[12] Herbert B.M., Pollatos O. (2012). The Body in the Mind: On the Relationship Between Interoception and Embodiment. Top. Cogn. Sci..

[13] Bruch H. (1982). Anorexia Nervosa: Therapy and theory. Am. J. Psychiatry.

[14] Treasure J., Cardi V. (2017). Anorexia Nervosa, Theory and Treatment: Where Are We 35 Years on from Hilde Bruch’s Foundation Lecture?. Eur. Eat. Disord. Rev..

[15] Fassino S., Pierò A., Gramaglia C., Abbate-Daga G. (2004). Clinical, Psychopathological and Personality Correlates of Interoceptive Awareness in Anorexia nervosa, Bulimia nervosa and Obesity. Psychopathology.

[16] Volpe U., Monteleone A.M., Monteleone P. (2018). Diagnostic Classification of Eating Disorders: The Role of Body Image. Body Image, Eating, and Weight.

[17] Borsboom D. (2017). A network theory of mental disorders. World Psychiatry.

[18] Borsboom D., Cramer A.O.J. (2013). Network analysis: An integrative approach to the structure of psychopathology. Ann. Rev. Clin. Psychol..

[19] Costantini G., Epskamp S., Borsboom D., Perugini M., Mõttus R., Waldorp L.J., Cramer A.O.J. (2015). State of the aRt personality research: A tutorial on network analysis of personality data in R. J. Res. Pers..

[20] Olatunji B.O., Levinson C., Calebs B. (2018). A network analysis of eating disorder symptoms and characteristics in an inpatient sample. Psychiatry Res..

[21] Elliott H., Jones P.J., Schmidt U. (2018). Central Symptoms Predict Post-Treatment Outcomes and Clinical Impairment in Anorexia Nervosa: A Network Analysis in a Randomized-Controlled Trial. PsyArXiv Preprints.

[22] Monteleone A.M., Cascino G., Solmi M., Pirozzi R., Tolone S., Terracciano G., Parisi S., Cimino M., Monteleone P., Maj M. (2019). A network analysis of psychological, personality and eating characteristics of people seeking bariatric surgery: Identification of key variables and their prognostic value. J. Psychosom. Res..

[23] Castellini G., Trisolini F., Ricca V. (2014). Psychopathology of eating disorders. J. Psychopathol..

[24] First M.B., Williams J.B.W., Karg R.S., Spitzer R.L. (2015). User’s Guide for the Structured Clinical Interview for DSM-5 Disorders, Research Version (SCID-5-RV).

[25] Garner D.M. (1991). Eating Disorder Inventory—2 Manual.

[26] Epskamp S., Cramer A.O.J., Waldorp L.J., Schmittmann V.D., Borsboom D. (2012). qgraph: Network Visualizations of Relationships in Psychometric Data. J. Stat. Softw..

[27] Epskamp S., Fried E.I. (2018). A tutorial on regularized partial correlation networks. Psychol. Methods.

[28] Friedman J., Hastie T., Tibshirani R. (2014). Glasso: Graphical Lasso-Estimation of Gaussian Graphical Models.

[29] Chen J., Chen Z. (2008). Extended Bayesian information criteria for model selection with large model spaces. Biometrika.

[30] Foygel R., Drton M. (2010). Extended Bayesian information criteria for Gaussian graphical models. Adv. Neural Inf. Proc. Syst..

[31] Opsahl T., Agneessens F., Skvoretz J. (2010). Node centrality in weighted networks: Generalizing degree and shortest paths. Soc. Netw..

[32] Epskamp S., Borsboom D., Fried E.I. (2018). Estimating psychological networks and their accuracy: A tutorial paper. Behav. Res. Methods.

[33] Fried E.I., Cramer A.O.J. (2017). Moving Forward: Challenges and Directions for Psychopathological Network Theory and Methodology. Perspect. Psychol. Sci..

[34] Forbes M.K., Wright A.G.C., Markon K.E., Krueger R.F. (2019). The network approach to psychopathology: Promise versus reality. World Psychiatry.

[35] Oldershaw A., Startup H., Lavender T. (2019). Anorexia Nervosa and a lost emotional self: A psychological formulation of the development, maintenance, and treatment of Anorexia Nervosa. Front. Psychol..

[36] Monteleone A.M., Mereu A., Cascino G., Criscuolo M., Castiglioni M.C., Pellegrino F., Patriciello G., Ruzzi V., Monteleone P., Vicari S. (2019). Re-conceptualization of anorexia nervosa psychopathology: A network analysis study in adolescents with short duration of the illness. Int. J. Eat. Disord..

[37] Markus H., Smith J., Moreland R.L. (1985). Role of the self-concept in the perception of others. J. Pers. Soc. Psychol..

[38] Nordbø R.H.S., Espeset E.M.S., Gulliksen K.S., Skårderud F., Holte A. (2006). The meaning of self-starvation: Qualitative study of patients’ perception of anorexia nervosa. Int. J. Eat. Disord..

[39] Skårderud F. (2007). Eating one’s words, Part II: The embodied mind and reflective function in anorexia nervosa—Theory. Eur. Eat. Disord. Rev..

[40] Jenkinson P.M., Taylor L., Laws K.R. (2018). Self-reported interoceptive deficits in eating disorders: A meta-analysis of studies using the eating disorder inventory. J. Psychosom. Res..

[41] Keizer A., Smeets M.A.M., Dijkerman H.C., van den Hout M., Klugkist I., van Elburg A., Postma A. (2011). Tactile body image disturbance in anorexia nervosa. Psychiatry Res..

[42] Zucker N., Moskovich A., Bulik C.M., Merwin R., Gaddis K., Losh M., Piven J., Wagner H.R., LaBar K.S. (2013). Perception of affect in biological motion cues in anorexia nervosa. Int. J. Eat. Disord..

[43] Keel P.K., Dorer D.J., Franko D.L., Jackson S.C., Herzog D.B. (2005). Postremission Predictors of Relapse in Women with Eating Disorders. Am. J. Psychiatry.

[44] Calugi S., Dalle Grave R. (2019). Body image concern and treatment outcomes in adolescents with anorexia nervosa. Int. J. Eat. Disord..

[45] Caglar-Nazali H.P., Corfield F., Cardi V., Ambwani S., Leppanen J., Olabintan O., Deriziotis S., Hadjimichalis A., Scognamiglio P., Eshkevari E. (2014). A systematic review and meta-analysis of ‘Systems for Social Processes’ in eating disorders. Neurosci. Biobehav. Rev..

[46] Treasure J., Schmidt U. (2013). The cognitive-interpersonal maintenance model of anorexia nervosa revisited: A summary of the evidence for cognitive, socio-emotional and interpersonal predisposing and perpetuating factors. J. Eat. Disord..

[47] Monteleone A.M., Treasure J., Kan C., Cardi V. (2018). Reactivity to interpersonal stress in patients with eating disorders: A systematic review and meta-analysis of studies using an experimental paradigm. Neurosci Biobehav. Rev..

[48] Biegel G.M., Brown K.W., Shapiro S.L., Schubert C.M. (2009). Mindfulness-based stress reduction for the treatment of adolescent psychiatric outpatients: A randomized clinical trial. J. Consult. Clin. Psychol..

