# Supplementary figures and images for: The Role of the Embodiment Disturbance in the Anorexia Nervosa Psychopathology: A Network Analysis Study
Source: Brain Sci. 2019 Oct 15;9(10):276. doi: 10.3390/brainsci9100276 (PMC6826416; doi:10.3390/brainsci9100276)

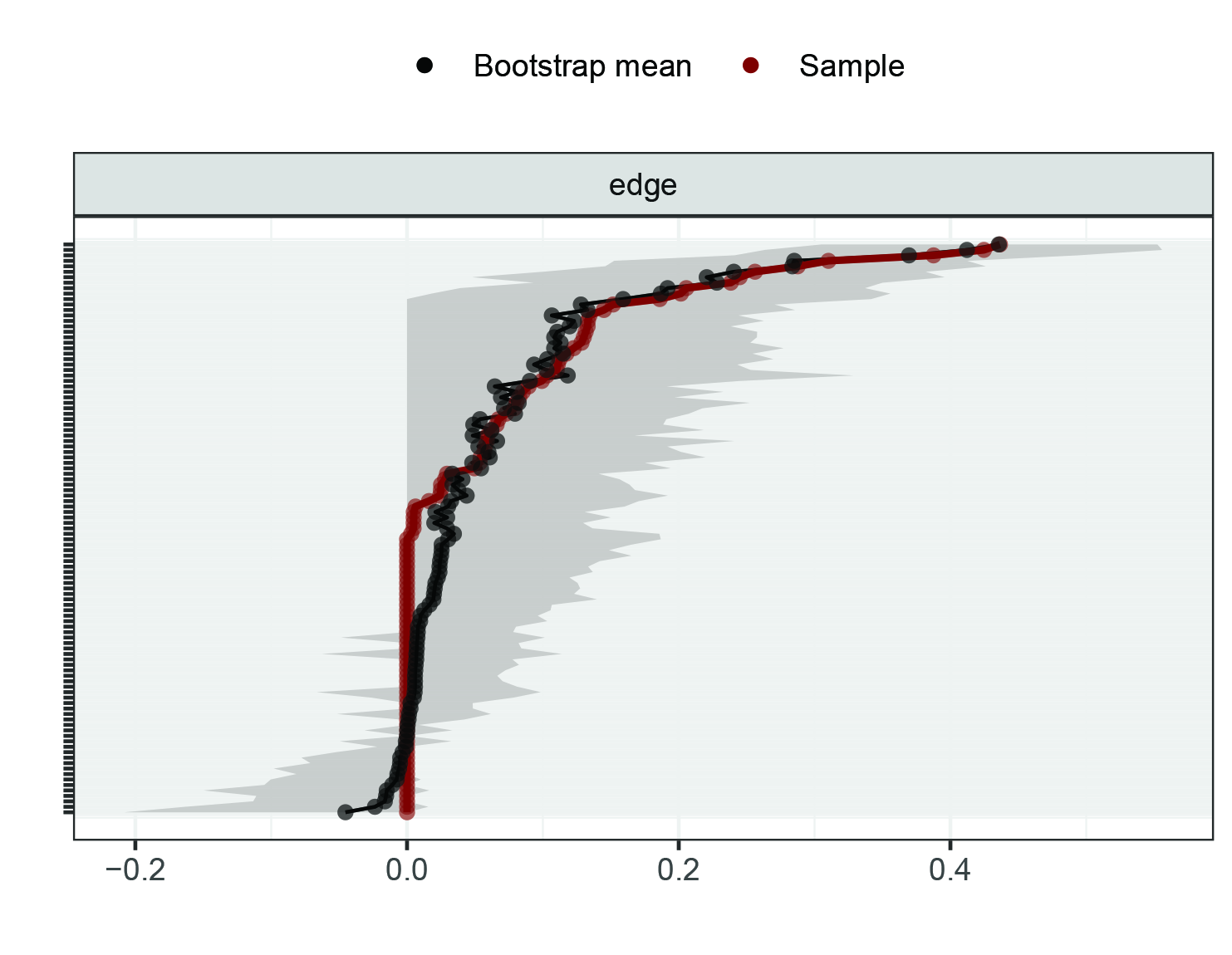

Supplement: Supplementary file 1 [file brainsci-09-00276-s001.zip › Supplementary Figure 2.tif]

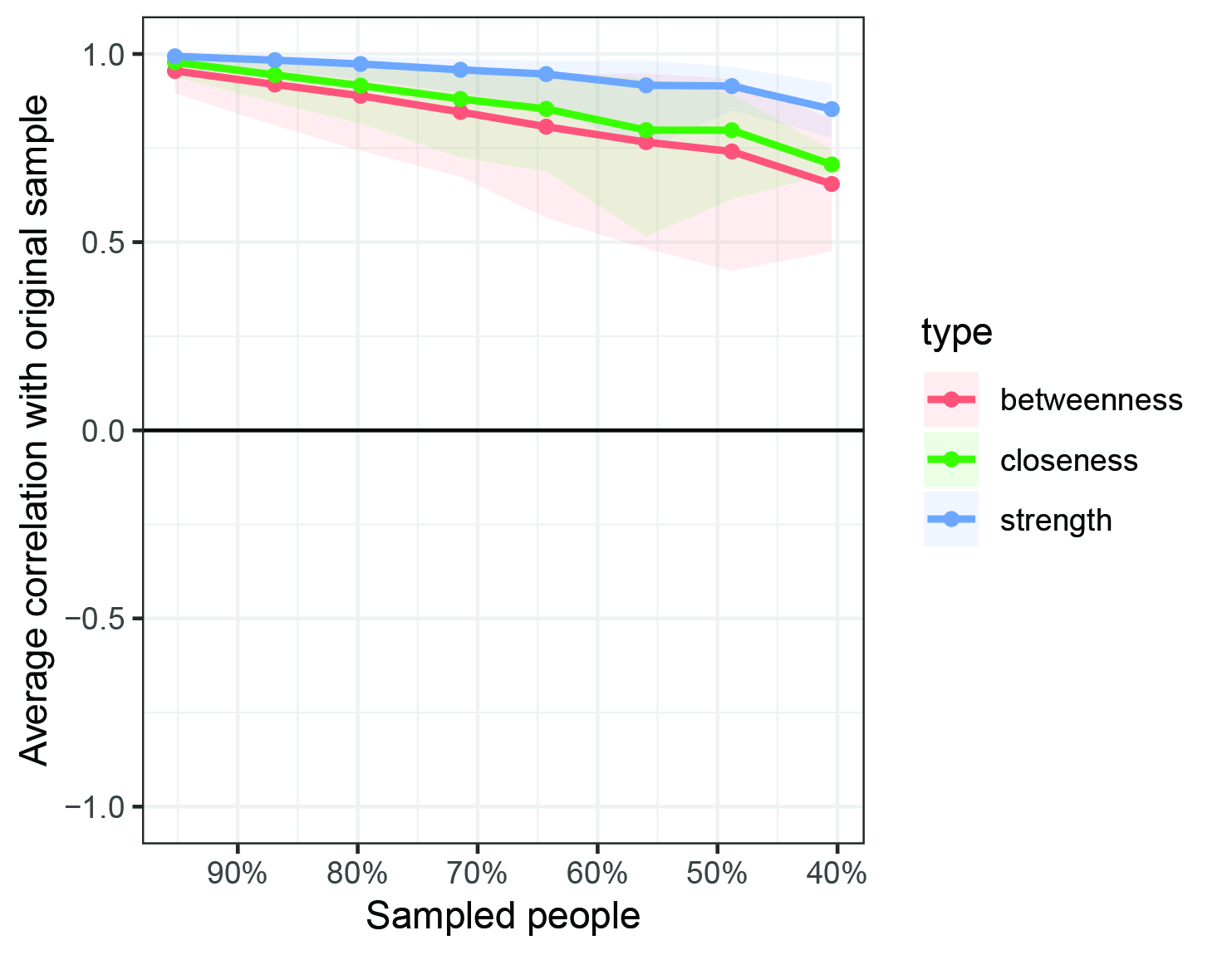

Supplement: Supplementary file 1 [file brainsci-09-00276-s001.zip › Supplementary Figure 1.tif]
